# Immobilization of gold nanoclusters inside porous electrospun fibers for selective detection of Cu(II): A strategic approach to shielding pristine performance

**DOI:** 10.1038/srep15608

**Published:** 2015-10-22

**Authors:** Anitha Senthamizhan, Asli Celebioglu, Brabu Balusamy, Tamer Uyar

**Affiliations:** 1 UNAM-National Nanotechnology Research Center, Bilkent University, Ankara, 06800, Turkey; 2 Institute of Materials Science & Nanotechnology, Bilkent University, Ankara, 06800, Turkey

## Abstract

Here, a distinct demonstration of highly sensitive and selective detection of copper (Cu^2+^) in a vastly porous cellulose acetate fibers (pCAF) has been carried out using dithiothreitol capped gold nanocluster (DTT.AuNC) as fluorescent probe. A careful optimization of all potential factors affecting the performance of the probe for effective detection of Cu^2+^ were studied and the resultant sensor strip exhibiting unique features including high stability, retained parent fluorescence nature and reproducibility. The visual colorimetric detection of Cu^2+^ in water, presenting the selective sensing performance towards Cu^2+^ ions over Zn^2+^, Cd^2+^ and Hg^2+^ under UV light in naked eye, contrast to other metal ions that didn’t significantly produce such a change. The comparative sensing performance of DTT.AuNC@pCAF, keeping the nonporous CA fiber (DTT.AuNC@nCAF) as a support matrix has been demonstrated. The resulting weak response of DTT.AuNC@nCAF denotes the lack of ligand protection leading to the poor coordination ability with Cu^2+^. The determined detection limit (50 ppb) is far lower than the maximum level of Cu^2+^ in drinking water (1.3 ppm) set by U.S. Environmental Protection Agency (EPA). An interesting find from this study has been the specific oxidation nature between Cu^2+^ and DTT.AuNC, offering solid evidence for selective sensors.

A major global threat emerged from water pollution caused by heavy metal ions have long been a subject of concern. Still, it continues to thrive as a challenging problem throughout the world^1^. Also, the release of copper ions into the environment has increased the need for a potential system owing to emergence of health hazards associated with the entry of toxic components into the food chain. This is further expected to cause severe neurodegenerative diseases, although it has a pivotal role in various physiological processes. According to the U.S. Environmental Protection Agency (EPA), the safety limit of copper in drinking water has been set as 1.3 ppm (16 μM)^2^,^3^. Therefore, a need for considerable effort towards the development of sensitive and selective sensor for on-site visual detection is the want of the hour. In recent years, major study has gone into the analysis of fluorescence based sensors owing to their high sensitivity, ease of operation, time-efficiency and sensitivity characteristics^4^,^5^,^6^,^7^,^8^. Moreover, significant progress has also been made to develop a variety of fluorescent probes and subsequent color changes with their aggregation in the presence of analytes has been investigated and studied in the recent past^9^,^10^,^11^.

The metal clusters engraved with the ligand are considered as a competent area of study, owing to their highly sensitive fluorescent nature towards changes in the microenvironment^12^,^13^,^14^. A key significance is sought towards practical applications in the adaptation of such efficient probes onto the solid support due to their convenient usage in the field. Yet, there still exists a strong demand in developing such systems for day-to-day applications^15^,^16^,^17^,^18^,^19^,^20^. Apart from sensitivity, a selective mechanism is generally employed by the presence of surface functional groups to interact with target analytes. However, limitation occurs in the real applicability of such systems due to the delicate nature of ligands and their inadequate performance on solid support. The combined protection of probes along with the ligand has proved it too difficult to evaluate. Still, most of these fluorescent based sensors have been employed in aqueous solution medium, significantly restricting their practical applications. Thus, a high demand for devising a stable and easily used platform for sensing is needed. This has involved the usage of the synergistic properties of fluorescent probe and solid support. Practically, the capacity of solid substrate for adsorbing metal ions can determine the sensitivity of probe in sensing performance. As one kind of solid support, paper-based sensors are taken into study for their disposable and on-site analysis capabilities^21^,^22^,^23^,^24^.

Unfortunately, limited surface area of such substrates and the need for large quantities of material to coat a small piece of area has constrained further application. A well proven fact is that the facilitation of electrospun fibers forms a stable platform for holding and protecting various functional materials and further enhances their properties in various applications^25^,^26^,^27^,^28^,^29^,^30^,^31^,^32^,^33^,^34^,^35^,^36^. In addition, various ligands have also been effectively grafted on fiber surface to sustain/enhance the attachment of nanoparticles^37^,^38^,^39^. In recent past, extensive application of nanofibers has been carried out owing to their large surface area, high porosity and ability to successfully functionalize on the surface^40^,^41^,^42^,^43^,^44^,^45^,^46^,^47^,^48^. Though there still exists few constrains regarding their stability and protection of ligands, further consideration is imperative to exploit them successfully into onsite applications.

The demand for Cu^2+^ specific visual sensor when coupled with our expertise in the design of electrospun fibers has prompted us to investigate the highly stable colorimetric sensor. We here describe an example of enhanced protection of dithiothreitol (DTT) capped gold nanoclusters (DTT.AuNC) encapsulated porous cellulose acetate fibers (pCAF) and their Cu^2+^ sensing performance. The realization of enhanced protection of DTT.AuNC in pCAF is achieved by the characteristic of higher surface area and porous nature of the CAF. The fluorescence nature of the DTT.AuNC in pCAF is highly sustained in the parent form without aggregation and any of their environmental limitations. Interestingly, a valuable advantage of the nanofibers as a solid support system is the ready incorporation of fluorescent probes into cavity while still retaining their distinct properties. Unlike the surface decorated DTT.AuNC on the nonporous cellulose acetate fiber (nCAF), the incorporation of these nanofibers imparts long-term stability of the DTT.AuNC. This also enhances facile accessibility of the adsorbed fluorescence probe present inside the porous structure to analytes, derived from the uniform distribution and incorporation of DTT.AuNC inside pCAF.

## Results

### Fabrication and characterization of DTT.AuNC incorporated porous cellulose acetate fibers (DTT.AuNC@pCAF)

**Figure 1:**
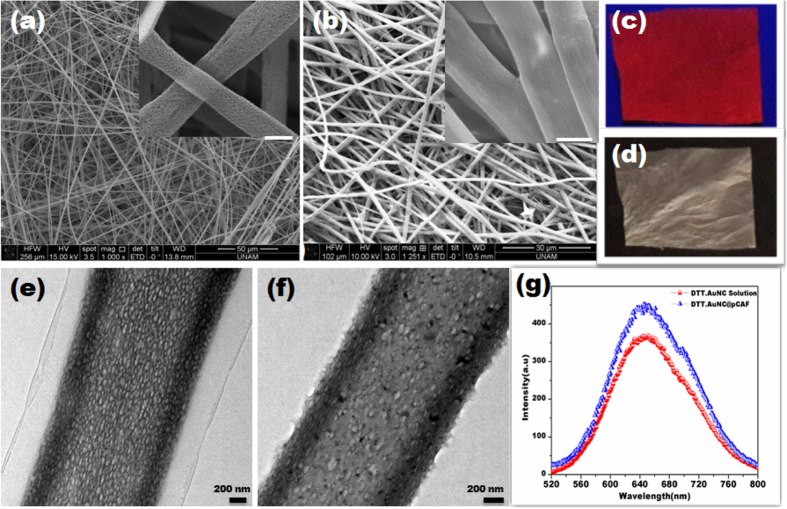
(**a**) SEM images of the electrospun pCAF before and (**b**) after incorporating DTT.AuNC. The higher magnification view shows as an inset in their corresponding figures (scale bar-1 μm). The image evidently confirms the porous structure of CAF and subsequently porous nature of CAF is reduced after incorporating DTT.AuNC (**c**) Photograph of the DTT.AuNC@pCAF under UV light (λ_ext_-254 nm) and (**d**) day light condition. TEM image of single DTT.AuNC@pCAF (**e**) in the presence and (**f**) absence of excess DTT ligand. (**g**) Compared emission spectra of DTT.AuNC solution and DTT.AuNC@pCAF.

The pCAF having an average diameter of 1 to 1.5 μm with pore diameter of 30–40 nm were fabricated using simple and versatile electrospinning technique. Evident facts were observed from the SEM image that porous structure of CAF resulted in rapid evaporation of highly volatile solvent and a subsequent solidification of CA polymer chains during the electrospinning process as depicted in Fig. 1a^49^. Based on reported procedure, the DTT capped gold nanoclusters (DTT.AuNC) were prepared^50^, and subsequently incorporated into the pCAF, named as DTT.AuNC@pCAF by using dip coating procedure. TEM image of the prepared AuNC and their size distribution diagram are shown in Fig. S1. The mean diameter of the AuNC was determined to be 2.5 ± 0.5 nm. Upon ultra violet (UV) light irradiation (λ_ext_-254 nm), the characteristics fluorescence nature of the DTT.AuNC solution has emitted intense red color as shown in Fig. S2. We further performed the XPS to confirm the encapsulation of AuNC inside the pCAF and to know the oxidation states of gold (Au) and sulphur (S). The observed Au (4f) spectrum composed of Au 4f_7/2_ and Au 4f_5/2_ peaks located at the binding energies of 84.2 and 87.8 eV, respectively which confirms the presence of both Au^+^ and Au^0^ as shown in Fig. S3. The S 2p curve is fitted into two peaks at 162.9 and 164.2 eV. The observed peaks are assigned to the formation of S-Au covalent bonds and free thiols/disulfide formation, respectively.

**Figure 2:**
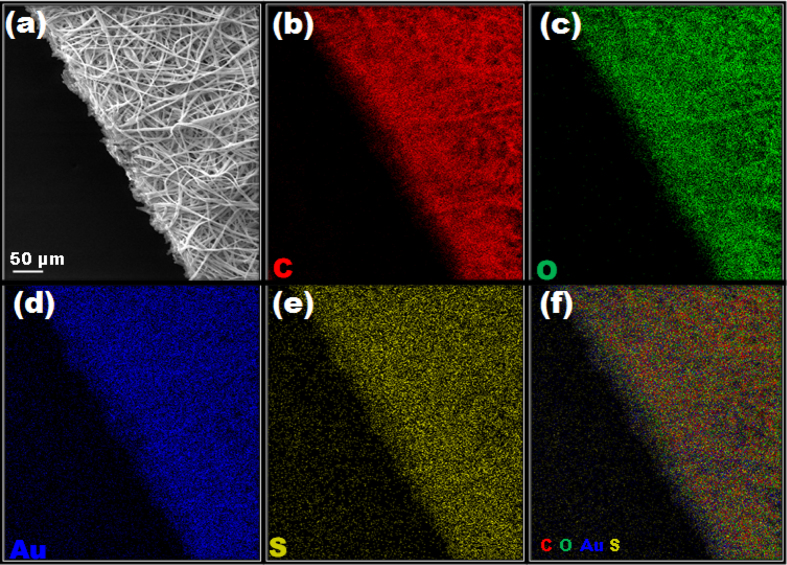
(**a**) SEM image of DTT.AuNC@pCAF and their corresponding (**b–e**) elemental mapping images of carbon, oxygen, gold, and sulfur, respectively. (**f**) overlay image of all elements.

Apparently, the existing pores in the resultant DTT.AuNC@pCAF didn’t get destroyed, with eventual decline in the size and number of the pores, as evident from Fig. 1b,f. Similar to the solution state, the retained parent fluorescence nature of DTT.AuNC inside the pCAF emits red color as shown in Fig. 1c,d. As observed in the TEM image (Fig. 1e), it is unambiguously demonstrated that deep penetration of DTT.AuNC into the interior of the fiber along with their coating density is independent of depth. Even after incorporation of DTT.AuNC, the diameter and structure of the pCAF does not cogently deteriorate due to the diffusion nature of AuNC inside the porous structure (~200 nm). Expressively, this implies the promotion of this method for incorporation rather than mere adsorption. The encapsulated DTT.AuNC in pCAF is uniform all over the fibrous membrane as evidenced from the elemental mapping shown in Fig. 2. Based on the Fig. S4, the flexible nature of the resultant DTT.AuNC@pCAF membrane affirms their adaptability into field applications similar to paper based sensors.

It is highly predictable that the hydrophobicity of most of the polymer based fiber matrix decrease the diffusion of analytes inside the fiber matrix and further contact with the probes results in lowering the sensing performance. To overcome this limitation, we have adopted dual functional pCAF which involves the utilization of stable and enhanced interaction with water as a support system for our sensing experiment. The swelling nature of pCAF in water facilitates extensive infiltration of DTT.AuNC into the interiors of the fibers, deriving improvement in the extent of incorporation amount inside the fiber. In addition, the concurrent evaporation of solvent contracting the pores and effectively trapping DTT.AuNC.

The anchoring mechanism for DTT.AuNC on surface of pCAF might be due to the formation of hydrogen bonding between the thiol (-SH) or hydroxyl group in DTT with acetate group in CA. As we expected, excess ligands are clearly evident on the surface of the fiber, thereby we remove the excess ligand by treating the fibers in water for 10 minutes with shaker. This has unveiled a noticeable difference, confirmed by the TEM image as shown in Fig. 1e,f. Additionally, the excess amount of ligands (see Fig. S5a,b) as well as the aggregated AuNC on the surface of the fiber may show a decrease in further interpreting the analytes and their connectivity, leading to decreased sensing performances. Strikingly, the extended period of coating (more than 3 hours) enables the aggregation of AuNC outside the pores of the pCAF as clearly illustrated in Fig. S5c,d.

Initially, it is pivotal to overcome two bottlenecks for the effective incorporation of DTT.AuNC onto pCAF. (I) Aggregation of DTT.AuNC on the fiber matrix. (II) Preservation of parent fluorescence of DTT.AuNC. Till date, the unpredictable and variable spectroscopic properties proven to be a major shortcoming of solid supported AuNC^43^. A presentation of the comparison between the fluorescence nature of the resultant DTT.AuNC@pCAF and the parent DTT.AuNC can be observed in Fig. 1g. Nevertheless, it is riveting to witness the unchanged fluorescence spectra of DTT.AuNC@pCAF, implying the homogenous dispersion of AuNC in which each pore act as a cavity to protect them, and not mere embedment. The effective encapsulation of DTT.AuNC into the porous fiber network not only offers a sensing platform but also enhances the stability of DTT.AuNC against environmental factors. For a period of more than six months, exceptional stability of DTT.AuNC@pCAF at normal atmospheric condition was observed without the decline of fluorescence emission intensity.

It is of prominent notice here that the incorporated DTT.AuNC does not get detached from the surface and also it is found that the coating density remains unaltered for a long time at room temperature. To confirm this particular structure, the DTT.AuNC@pCAF were exposed to water for nearly ten days and the corresponding results were monitored in terms of their visual identification (Fig. S6) and their relative fluorescence intensity (Fig. S7). This strongly entails the mechanism behind their stability to be because of the deep penetration of DTT.AuNC into the pCAF and subsequent protection by the porous cavity. It is found that even after prolonged exposure to water, the membrane doesn’t get defaced and no detectable pieces are observed in water, as can be seen in Fig. S8. In order to compare their stability and confirm their usability in warm countries, the DTT.AuNC@pCAF was exposed to 50˚C and as anticipated the membrane is found to be more stable and possess well maintained fluorescence characteristics (Fig. S9).

In addition, we have considered DTT.AuNC decorated nonporous cellulose acetate fibers (DTT.AuNC@nCAF) as a control to highlight the advantages of using a porous structure in sensing performance. The SEM image of nCAF before and after decorating the DTT.AuNC are shown in Fig. S10 and the inset represents their photograph under UV light (λ_ext_-254 nm). Stressing on its stability, a decrease in fluorescence intensity during their treatment with water resulted in the detachment of DTT.AuNC from the surface of the fibers as shown in Fig. S7.

### Sensing performance

Effective contact mode has been used as the approach to display the visual colorimetric response of the sensor strip in the presence of Cu^2+^. To evaluate the sensing performance, a piece of DTT.AuNC@pCAF membrane was immersed into various Cu^2+^ concentrations for 10 minutes separately and then dried at room temperature. To explore its potentiality in serving as an environmental-friendly platform, its stability against water has been analyzed as primary agenda. As means to validate this fact, the membrane strip was tested with water in the absence of Cu^2+^ to verify their sensitivity either towards analytes or water. The obtained results denoted no significant color change when viewed by naked eye under UV light (λ_ext_-254 nm) as well as under normal light conditions.

**Figure 3:**
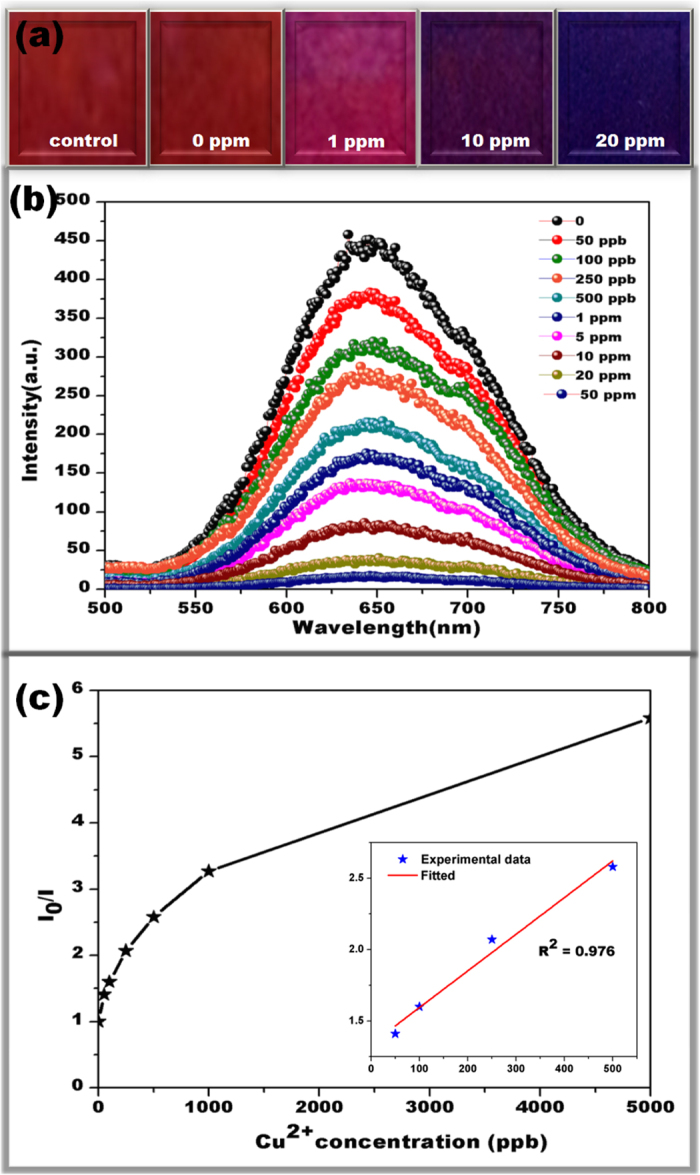
(**a**) Visual colorimetric detection of Cu^2+^. A clear difference in the color from red to blue is noticed under UV light with increasing concentration of Cu^2+^. The photograph has been taken under exposure of UV light (λ_ext_-254 nm). (**b**) Fluorescence spectra of DTT.AuNC@pCAF upon exposure to various concentration of Cu^2+^ in water and their (**c**) relative fluorescence intensity variation (I_0_/I where I_0_ and I are the emission intensity of DTT.AuNC@pCAF in the absence and presence of different concentration of Cu^2+^). The results demonstrated the gradual quenching of fluorescence intensity upon increasing the concentration of Cu^2+^. The excitation wavelength was set as 280 nm.

Interestingly, upon increasing the Cu^2+^ concentration, a significant and uniform color change from red to blue has been noticed with the visual detectable limit of 1 ppm (16 μM) as clearly illustrated in Fig. 3a. It is also clear that this is lower than the maximum level (1.3 ppm or 20 μM) of Cu^2+^ in drinking water permitted by the U.S. EPA^2^. To elucidate the efficiency of the porous structure for sensing performance, we have studied and compared the sensing responses with DTT.AuNC@nCAF (see Fig. S11). The resulting weak response denotes the lack of ligand protection leading to the poor coordination ability with Cu^2+^. Therefore, we concluded that the observed sensing performance in the porous structure might resulted from higher diffusion ability of Cu^2+^ into the interior surface of the fibrous membrane, followed by their uniform response in the emission intensity throughout the membrane strip.

The designed sensor strip here is applicable for visual colorimetric detection without the aid of any complicated instruments and can be applied for outdoor applications. Further, the fluorescence spectra of DTT.AuNC@pCAF have been recorded upon addition of various concentration of Cu^2+^. The relative fluorescence intensity variation (I_0_/I where I_0_ and I are the emission intensity of DTT.AuNC@pCAF in the absence and presence of different concentration of Cu^2+^ respectively) as a function of Cu^2+^ concentration has clearly demonstrated the gradual quenching upon increasing the concentration of Cu^2+^ (Fig. 3b,c) and lowering the limit of detection to 50 ppb.

### Interference studies

**Figure 4:**
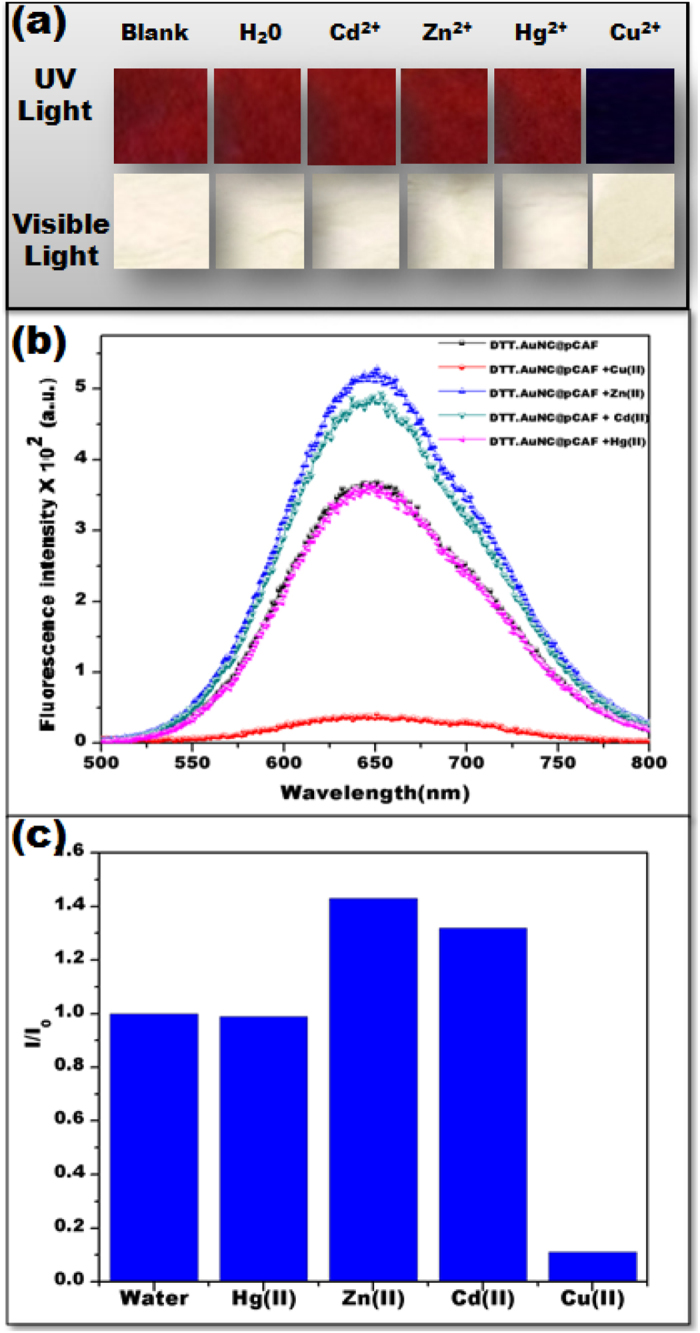
Selective visual colorimetric response of Cu^2+^. (**a**) Photograph of DTT.AuNC@pCAF treated with different metal ions at 20 ppm concentration and their (**b**) corresponding emission spectra. The image evidently confirms the dramatic fluorescence quenching upon addition of Cu^2+^, whereas no detectable changes were detected in water as well as other interfering metal ions. (**c**) Plot of I_0_/I against Cu^2+^ with the competent metal ions.

To examine further specificity of DTT.AuNC@pCAF as a copper selective sensor, the fluorescence quenching behavior of DTT.AuNC@pCAF against various metal ions including Hg^2+^, Cd^2+^ and Zn^2+^ were tested under same conditions at a concentration of 20 ppm. The selective visual colorimetric response is presented in Fig. 4a. As can be studied from the image, dramatic fluorescence quenching has been obtained upon addition of Cu^2+^, whereas no detectable changes were detected in water as well as other interfering metal ions. Further, similar results from fluorescence spectra have been attained, where no changes to the fluorescence behavior was caused by Hg^2+^, whereas enhanced fluorescence intensity has been observed in cases of Zn^2+^ and Cd^2+^ which might be due to the formation of zinc and cadmium complex with DTT as shown in Fig. 4b,c. Nevertheless, the enhancement in their fluorescence was not visible to naked eye under UV light (λ_ext_-254 nm).

### Mechanism for selective sensing

**Figure 5:**
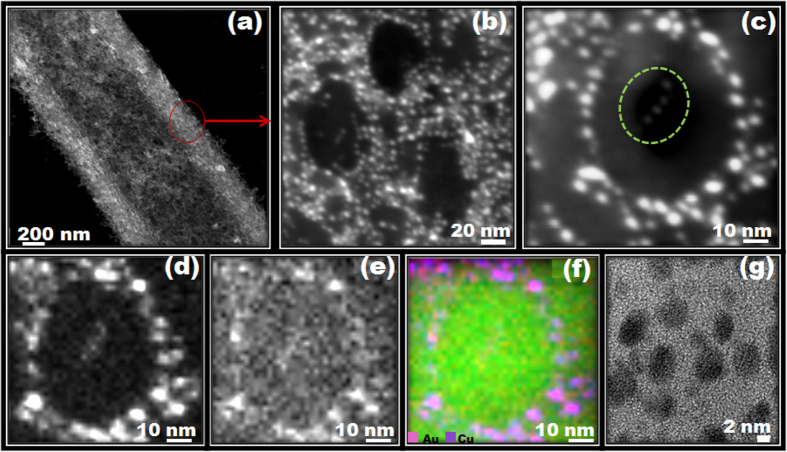
(**a–c**) HAADF-STEM (high-angle annular dark-field scanning transmission electron microscopy) image of Cu^2+^ treated DTT.AuNC@pCAF at different magnification. STEM-EDX elemental mapping of (**d**) Au and (**e**) Cu of formed Au–Cu blend. (**f**) The overlapped mapping images of d and e. (**g**) HRTEM image of Au–Cu blend.

As we had anticipated, the sensing performance of the DTT.AuNC@pCAF was found to be higher than that of the DTT.AuNC@nCAF. The key mechanism is the ability of the porous structure to increase the diffusion of the analytes inside the fibers and facilitate their complexation efficiency^51^,^52^,^53^. Secondly, the uniformly distributed DTT.AuNC inside the porous structure supplements the reactive sites for Cu^2+^ and thus higher sensitivity is attained. An interesting find from this technique has been the rapid and uniform adsorption of copper ions on the gold surface owing to the strong interaction with DTT, which has also been confirmed by TEM analysis as clearly shown in Fig. 5. Provocatively, as long as the coating density is made available, pores in the pCAF act as a channel through which the analytes easily enter the fiber, except a few particles which are seen inside the pores. As a result, the copper ions adsorbed around the gold cluster have been confirmed by HAADF-STEM (high-angle annular dark-field scanning transmission electron microscopy) image and mapping of gold and copper has been depicted in Fig. 5a–g. Further, we noticed the aggregated Au-Cu blend upon treatment with Cu^2+^. Moreover, the AuNC are not detached from the surface of the fibers even after strong interaction with Cu^2+^ and injured capping agent.

Detailing the structure of DTT, it is apparent that it contains two end thiol, one of which might be expected to attach with AuNC by strong covalent interaction between Au and –SH and the other –SH group remains free^54^,^55^. Furthermore, it has been established that the metal ions have the tendency to react with free thiol group and form complexes^56^. As indicated in the result, the decrease in fluorescence intensity could be due to the reducing energy transfer from the gold core to the metal ligand complex during effective oxidation of DTT by Cu^2+^. Affirming this mechanism, UV spectra have been recorded for Cu^2+^ treated DTT.AuNC and the broad absorption band around 270 nm indicated the characteristic band of oxidized DTT as shown in Fig. S12^57^.

**Figure 6:**
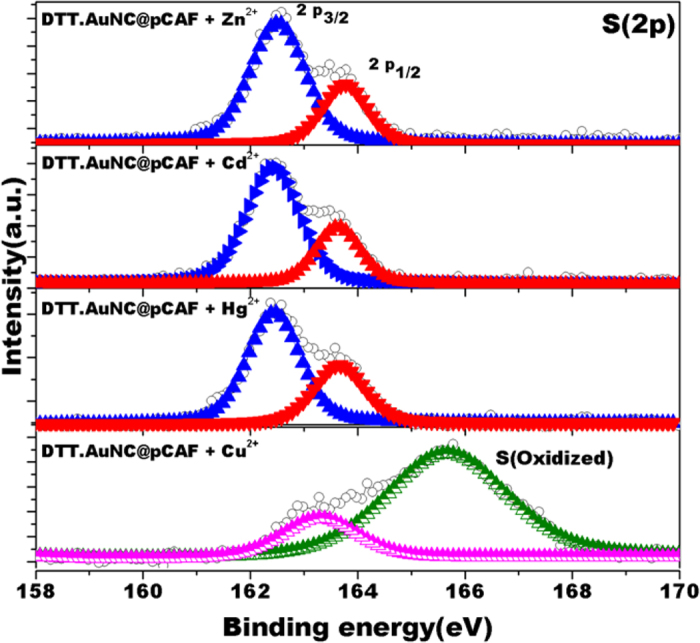
XPS spectra in the S(2p) region of the DTT.AuNC@pCAF after treatment with Zn^2+^, Cd^2+^, Hg^2+^and Cu^2+^.

As shown in Fig. S13, the binding energy of Au(4f) peak is shifted to higher energy level when treated with Cu^2+^ which implies that the oxidation of gold and there is no such shift was observed for other metal ions^58^,^59^ as confirmed from Fig. S13. In supporting our suspicion, we cleared on another possibility that DTT can reduce Cu^2+^ to Cu^+^ and further react with Au via 3d^10^(Cu^+^)-4d^10^(Au^+^) metallophilic interactions to induce fluorescence quenching^60^. Further, the oxidized sulphur is clearly visualized in the spectra as shown in Fig. 6. The specific interaction and oxidation nature between Cu^2+^ and DTT.AuNC is a proof of concept for selective sensor eventhough DTT have ability to bind with other metal ions. The presence of thiol group in DTT.AuNC might expected to make a bond and forms strong network with Zn^2+^ and Cd^2+^which could be the reason for enhanced fluorescence. To summarize, we strongly conceive that the coordination of Cu^2+^ in the presence of DTT around the AuNC is the cause for the occurrence of fluorescence quenching. Therefore, high recommendation is placed for the protection of the capping layer inside the cavity in order to sense the targeted analytes.

## Conclusions

To conclude, we have successfully presented the incorporation of fluorescent DTT capped AuNC onto the electrospun porous CA fibers and used them as a visual Cu^2+^ platform to detect concentration till 1 ppm visually. Interestingly, the porous structure has proved to be an effective platform for preserving the parent properties of DTT.AuNC. The obtained DTT.AuNC@pCAF membrane is found to possess high stability against environment, water and temperature. The underlying mechanism involves the combination of analyte-induced fluorescence quenching behavior of DTT.AuNC along with the unique features of electrospun nanofibers including enhanced surface area and porous structure. This procedure has been carried out by selective quenching of DTT.AuNC@pCAF in the presence of Cu^2+^, accompanied by a visual color change from red to blue. A systematic comparative sensing performance of DTT.AuNC@pCAF with DTT.AuNC@nCAF has been performed to elucidate the importance of porous structure in enhanced sensing performance. We have listed several unique characteristics and advantages of the prepared DTT.AuNC@pCAF: (1) selective detection of copper ions in water (2) highly stable sensor strip for outdoor application (3) no formation of toxic product after the sensing performances in water (4) easy handling and disposal. Therefore, we concluded that the observed sensing performance in the porous structure might resulted from higher diffusion ability of Cu^2+^ into the interior surface of the fibrous membrane, followed by their uniform response in the emission intensity throughout the membrane strip.

### Experimental details

#### Materials

Tetrachloroauric acid trihydrate (HAuCl_4_.3H_2_O), Dithiothreitol (DTT), dichloromethane (DCM, ≥99% (GC), Sigma-Aldrich), methanol, acetone (≥99% (GC), Sigma-Aldrich) cellulose acetate, (CA, Mw: 30000 g/mol, 39.8wt.% acetyl, Sigma-Aldrich), zinc acetate dihydrate (Sigma-Aldrich, ~98%), copper(II) acetate hydrate (Sigma-Aldrich, 98%), cadmium nitrate tetrahydrate (Fluka) and mercuric acetate (Merck) were purchased and used without any purification. Deionized water was used from a Millipore Milli-Q Ultrapure Water System.

#### Electrospinning of porous and nonporous CA fibers

A detailed description of the preparation of porous CA and nonporous CA fibers was presented in our previous study^49^. Initially, the homogenous electrospinning solution was prepared by dissolving CA in a DCM/acetone (1/1 (v/v)) binary solvent mixture at 10% (w/v) polymer concentration to obtain porous CA electrospun fibers. For nonporous CA nanofibers, the electrospinning solution was prepared by dissolving polymer in a DCM/methanol (4/1 (v/v)) binary solvent mixture at a 12% (w/v) polymer concentration. Subsequently, the clear solution was loaded in a 3 mL syringe fitted with a metallic needle of 0.4 mm inner diameter. This was followed by horizontal placement on a syringe pump (model KDS-101, KD Scientific, USA). The electrode of the high-voltage power supply (Spellman, SL30, USA) was clamped to the metallic needle, and the plate aluminum collector was grounded. The following electrospinning parameters were maintained: feed rate of solutions = 0.5–1 mL/h, applied voltage = 10–15 kV, tip-to-collector distance = 10–12 cm. The grounded stationary metal collector covered with an aluminum foil was used to deposit the electrospun CA fibers. The electrospinning apparatus was enclosed in a Plexiglas box and the process of electrospinning was carried out at 23°C at 18% relative humidity. The obtained fibers/webs were dried overnight at room temperature in a fume hood.

#### Synthesis of DTT capped gold nanoclusters (DTT.AuNC)

In light to a previously reported method, the DTT capped gold nanoclusters were prepared^50^. Initially, dissolution of DTT (11 mg) in 10 mL deionized water was done followed by vigorous stirring. This was continued with the addition of 768 μL of HAuCl_4_ solution. Exactly after three minutes, a solution of sodium hydroxide was added till pH of the solution reached 8. A colorless solution was formed, visible by the naked eye, followed by continuous stirring for a time period of 6 hours. Finally, under UV light (λ_ext_-254 nm), the prepared DTT capped AuNC emits red fluorescence, with subsequent incorporation into the electrospun fibers.

#### Preparation of DTT capped gold nanoclusters incorporated CA fibers (DTT.AuNC@pCAF and DTT.AuNC@nCAF )

The porous and nonporous CA fibrous membranes were soaked individually in a DTT.AuNC solution under shaking for three hours. Then the membrane was separated from the solution carefully and dried in air at room temperature. This was continued with washing the membrane under shaking for 15 minutes to remove excess ligands adsorbed on the surface of the membrane and further the membrane has been dried at room temperature. The porous and nonporous CA fiber samples named as DTT.AuNC@pCAF and DTT.AuNC@nCAF, respectively.

#### Analytical procedure

Preparation of a stock solution of 50 ppm Cu^2+^ was carried out by dissolving an appropriate amount of copper acetate in deionized water. Serial dilution of the stock solution was utilized in determining various concentrations. Similar procedure was followed for the preparation of ions including Cd^2+^, Zn^2+^ and Hg^2+^. For further characterization, a piece of membrane strip was immersed in the corresponding Cu^2+^ solutions and then dried at room temperature. Subsequential transitions in the fluorescence nature of the fibrous membrane have been observed under UV irradiation (λ_ext_-254 nm) and their fluorescence spectra.

#### Visual detection of Cu^2+^

For the visual detection of Cu^2+^, DTT.AuNC@pCAF membrane were cut into small pieces and immersed in different concentrations of Cu^2+^ solution for 10 minutes. After taking the membrane from the solution, it was dried at room temperature and exposed under UV light (λ_ext_-254 nm) to verify their color change, thereby collecting the fluorescence spectra. For all the other proposed metal ions, a similar procedure was followed and subsequent results were obtained.

#### Characterization

The morphology, diameter and porous nature of the electrospun fibers were analyzed by scanning electron microscope (SEM, Quanta 200 FEG) and transmission electron microscopy (TEM, Tecnai G2 F30) equipped with an EDS. And also, the distribution of gold nanocluster/metal ions on the fiber surface was studied by using STEM-EDX (scanning transmission electron microscopy-energy dispersive X-ray analysis). Fluorescence emission spectra were measured by time-resolved fluorescence spectrophotometer (FL-1057 TCSPC). The absorbance spectra were recorded using UV-Vis-NIR Spectrophotometer (Cary 100). The chemical composition and oxidation state of the elements in DTT.AuNC@pCA were studied by using X-ray photoelectron spectroscopy (XPS, Thermo K-alpha-monochromated).

## Additional Information

**How to cite this article**: Senthamizhan, A. *et al.* Immobilization of gold nanoclusters inside porous electrospun fibers for selective detection of Cu(II): A strategic approach to shielding pristine performance. *Sci. Rep.* **5**, 15608; doi: 10.1038/srep15608 (2015).

## Supplementary Material

Supplementary Information
