# Supplementary material for: Immobilization of gold nanoclusters inside porous electrospun fibers for selective detection of Cu(II): A strategic approach to shielding pristine performance
Source: Sci Rep. 2015 Oct 22;5:15608. doi: 10.1038/srep15608 (PMC4614998; doi:10.1038/srep15608)
Supplement: Supplementary Information [file srep15608-s1.doc]

**Supporting Information**

**Immobilization of gold nanoclusters inside porous electrospun fibers for selective detection of Cu(II): A strategic approach to shielding pristine performance**

*Anitha Senthamizhan,^*,†^ Asli Celebioglu, ^†,‡,#^ Brabu Balusamy,^†, #^ Tamer Uyar ^*,†, ‡^*

^†^UNAM-National Nanotechnology Research Center, Bilkent University, Ankara, 06800, Turkey

^‡^ Institute of Materials Science & Nanotechnology, Bilkent University, Ankara, 06800, Turkey

*^#^*Authors contributed equally

* [uyar@unam.bilkent.edu.tr](../../../../K:%5CManu%20V-All%20copper%20sensor%5Cuyar@unam.bilkent.edu.tr)

* [senthamizhan@unam.bilkent.edu.tr](../../../../K:%5CManu%20V-All%20copper%20sensor%5Csenthamizhan@unam.bilkent.edu.tr)

**Figure S1. (a)TEM image and (b) size distribution histogram of DTT capped gold nanoclusters.**

**Figure S2. Photographs of the DTT.AuNC solution exposed under (a) day light and (b) UV light (λ_ext_-254 nm).**

**Figure S3. XPS spectra of (a) Au(4f) and (b) S (2p) in DTT.AuNC@pCAF**

**
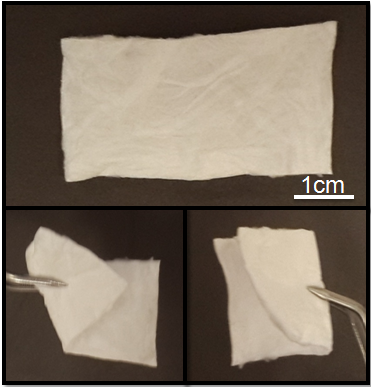
**

**Figure S4. Flexible nature of DTT.AuNC@pCAF.**

**
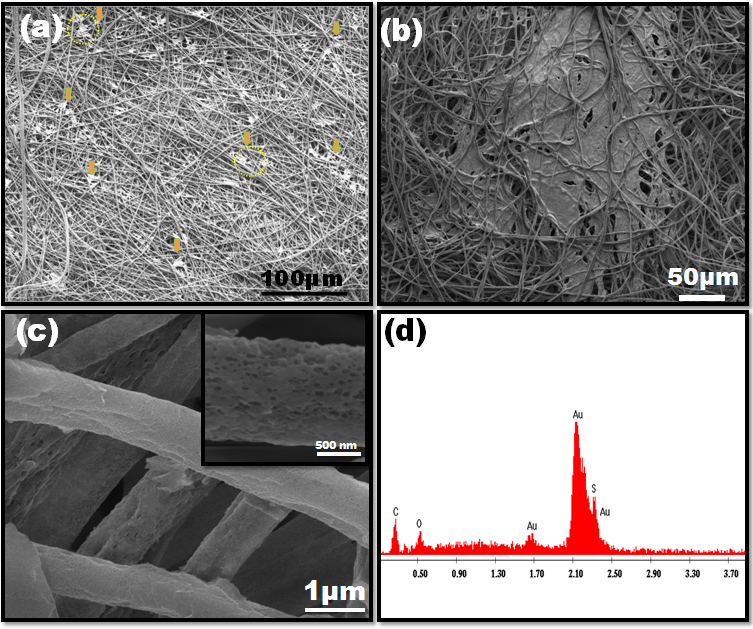
**

**Figure S5. (a-b) SEM images of DTT.AuNC@pCAF with excess ligand. (c) Effect of coating time on the incorporation of DTT.AuNC inside the pores (12 hours). The result clearly confirms the extended period of coating enables the aggregation of AuNC outside the pores of the pCAF (d) EDX spectra of aggregated AuNC on DTT.AuNC@pCA. The carbon (C) and oxygen (O) peaks are comes from the cellulose acetate.**

**Figure S6. Stability of DTT.AuNC@pCAF against water. The piece of DTT.AuNC@pCAF was exposed to water for 10 days and the photograph has been taken under UV light exposure at different time intervals.**

**Figure S7. Relative fluorescence intensity (I/I_0_) of DTT.AuNC@pCAF and DTT.AuNC@nCAF treated with water at different time intervals.**

**Figure S8. Photographs of DTT.AuNC@pCAF on (a) day 1 and (b) day 10 under normal light condition. The observed results suggested that the membrane doesn’t get defaced even after prolonged exposure to water.**

**Figure S9. Fluorescence spectra of DTT.AuNC@pCAF treated at different temperature.**

**
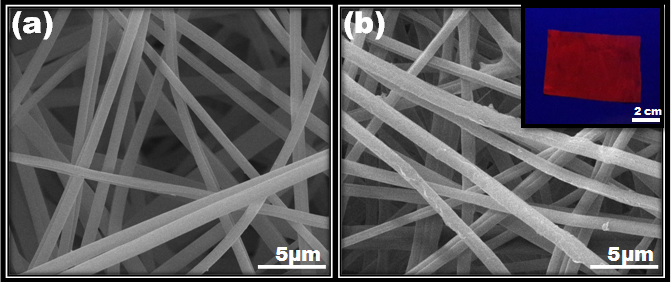
**

**Figure S10. SEM image of nonporous CA fiber (a) before and (b) after decorating DTT.AuNC. The inset shows the photograph of DTT.AuNC@nCAF upon exposure to UV light (λ_ext_-254nm).**

**Figure S11. Comparative sensing performance of (a) DTT.AuNC@pCAF with (b) DTT.AuNC@nCAF. The photograph has been taken under UV light (λ_ext_-254nm) in the presence of 1 ppm Cu^2+^ in water.**

**Figure S12. UV spectra of different metal ion treated DTT.AuNC at 20 ppm concentration.**

**
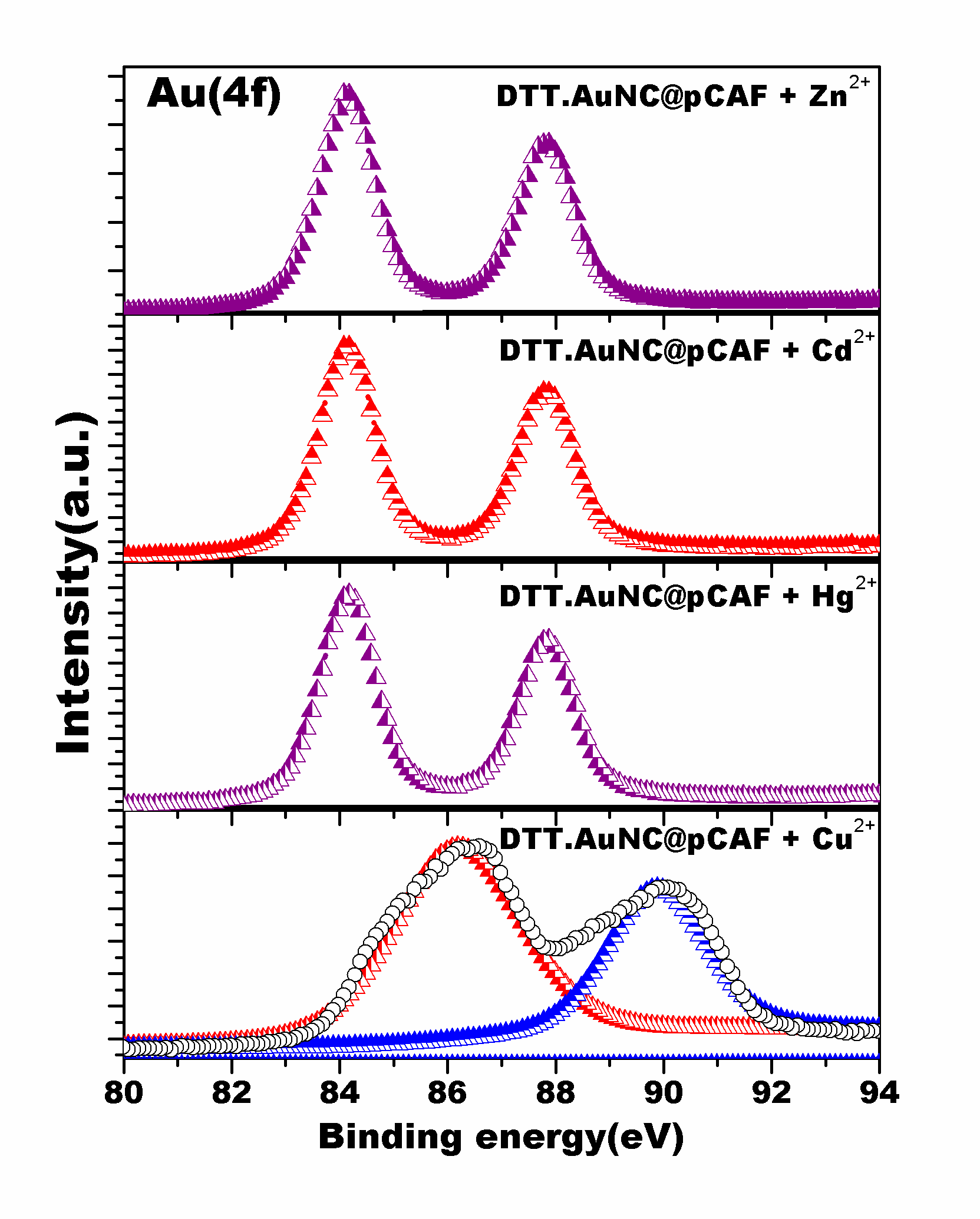
**

**Figure S13. XPS spectra in the Au(4f) region of DTT.AuNC@pCA after treatment with Zn^2+^, Cd^2+^, Hg^2+^and Cu^2+^.**
